# Diversifying Natural Products with Promiscuous Glycosyltransferase Enzymes via a Sustainable Microbial Fermentation Approach

**DOI:** 10.3389/fchem.2017.00110

**Published:** 2017-12-04

**Authors:** Ramesh P. Pandey

**Affiliations:** ^1^Department of BT-Convergent Pharmaceutical Engineering, Sun Moon University, Asan, South Korea; ^2^Department of Life Science and Biochemical Engineering, Sun Moon University, Asan, South Korea

**Keywords:** fermentation, natural products, glycosyltransferases, microbial engineering, sustainable method

Naturally occurring bacterial natural product (NP) glycosides are known to contain more than 344 distinct carbohydrate moieties in their structures and comprise ~21.5% of total secondary metabolites (Elshahawi et al., 2015). This diversity of NP-glycosides is generated by glycosyltransferases (GTs; EC 2.4), which are capable of harnessing and manipulating diverse donor and acceptor substrates. Although a high number of GTs have been identified and their sequences deposited to relevant databases, relatively few of them have been explored functionally, structurally, and mechanistically. Lack of structural and mechanistic insights into GTs is one of the major hurdles in engineering and applications of these promising enzymes in biotechnology.

Recent advances in system/synthetic biology, chemical, and metabolic engineering tools have opened up enormous opportunities to create NP-diversity by exploring different routes of NP biosynthesis and enzyme engineering (Kim E. et al., 2015; King et al., 2016; Smanski et al., 2016; Zhang et al., 2016). In addition, engineering of NPs tailoring enzymes that diversify parent NPs with tailored pharmacological properties are becoming a highly promising strategy to be used to modify therapeutically, cosmetically, and neutraceutical important molecules (Tibrewal and Tang, 2014; Antonopoulou et al., 2016; Pandey et al., 2016; Schmid et al., 2016).

Recently, GT, one of the NPs post-modifying enzymes, has been used for microbial biotransformation of valuable compounds in the production of diverse NP-glucosides and glycosides. Such strategies are based on the engineering of microbial cells, such as *Escherichia coli* and *Streptomyces*, for the production of a pool of different thymidine diphosphate (dTDP)/uridine diphosphate (UDP)-sugars in the cell cytosol, as shown in Figure 1 (Oh et al., 2007; Malla et al., 2013; Shinde et al., 2013; Song et al., 2013; Kim et al., 2017; Park et al., 2017). The central nucleotide diphosphate (NDP)-sugar biosynthetic pathways are engineered and diverted to produce the desired target NDP-sugars by heterologous expression of genes. To generate cytosolic pools of rare NDP-sugars that are usually not present in prokaryotic cells such as *E. coli*, the intermediate utilizing genes are either knock-out or repressed, while genes encoding all proteins of the entire NDP-sugar pathway are overexpressed. For instance, most of the rare microbial dTDP-sugars are biosynthesized via thymidine diphosphate 4-keto-4,6-dideoxy-d-glucose (dTKDG) and further modified by other consecutive sugar nucleotide-modifying enzymes that include epimerases, mutases, decarboxylases, oxidases, and reductases (Thibodeaux et al., 2008). These enzymes can cause substitutions and/or eliminations of various functional groups in the sugar moieties, such as changes in the methyl group (by sugar-*O*-methyltransferases, *N*-methyltransferases), amino group (by aminotransferases), sulfur group (by sulfotransferases), phosphate group (by phosphotransferases), as well as acyl and malonyl groups (by sugar acyl- and malonyl-transferases) (Tanner, 2001). In some instances, other bulky groups can be attached to NP-glycosides such as galloyl, prenyl, and long-chain fatty acyl side chains. Nucleotide sugar-modifying enzymes contribute to the diversification of NP-glycosides. dTKDG is one of the intermediates of differently activated nucleotide deoxy-sugars, which is present in *E. coli*. Thus, to divert the flow of dTKDG toward the target dTDP-sugar, genes in the dTKDG-consuming pathway need to be repressed. Such engineering approaches have recently been accomplished for the production of different NDP-sugars in *E. coli* (Kim B. G. et al., 2015; Pandey et al., 2015) and *Streptomyces* (Oh et al., 2007; Han et al., 2011, Shinde et al., 2013; Kim et al., 2017). These activated sugar moieties are eventually transferred to a diverse array of acceptor molecules via promiscuous GTs.

**Figure 1:**
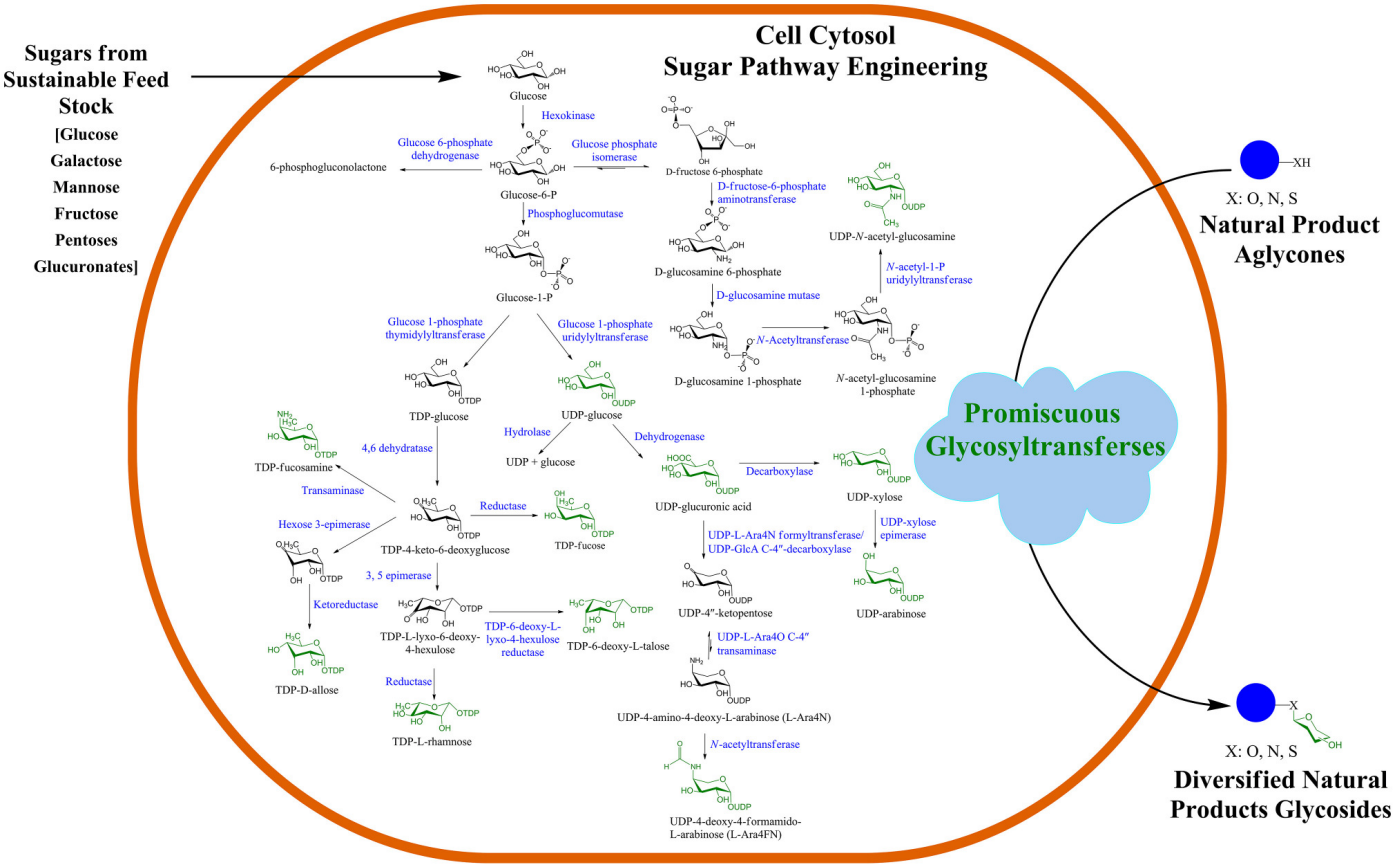
Microbial natural product (NP) glycosylation platform. GT-mediated *in vivo* glycosylation of exogenously supplemented diverse NPs in an engineered microbial cell. Such engineered microbial cells could be employed as cell factories for scale-up and industrial production of valuable glycosides.

The transfer of sugars from activated nucleotide sugar donors to acceptor molecules is mediated by GTs (Liang et al., 2015). Some of the GTs that are widely used to generate several NP-glycosides include OleD from *Streptomyces antibioticus* and its variants (Williams et al., 2007), *Bacillus* GTs (BcGTs, YjiC), plant GTs such as those from *Vitis vinifera* (VVGT1, VvGT5), *Arabidopsis thaliana* (ArGT-3), *Medicago trancatula* (UGT71G1, UGT85H2), and other bacterial GTs from several actinomycetes (GtfE, GalG1, UrdGT2, LanGT2) (Erb et al., 2009; Liang et al., 2015; Pandey et al., 2016). These GTs exhibit different degrees of substrate flexibility toward NDP-sugars and aglycons. Such promiscuous enzymes enable the process of diversification of NPs by conjugating a number of sugars to a broad range of aglycons while generating an array of structurally different natural and non-natural compounds (Thibodeaux et al., 2008; Gantt et al., 2011). However, other GTs have stringent specificity toward both donor and acceptor substrates, which remains a limiting factor in NP diversification. Thus, identification or generation of highly promiscuous GTs by either site-directed mutagenesis or domain-swapping approaches have been widely performed for *in vitro* production of glyco-randomized NPs (Fu et al., 2004; Zhang et al., 2006; Williams et al., 2008; Park et al., 2009; Gantt et al., 2013; Kim et al., 2013; Le et al., 2014; Parajuli et al., 2014; Liang et al., 2015). Some of the newly synthesized glycosides are found to exhibit potent biological activities with enhanced water solubility and bioavailability compared to the parent molecule.

Chemical synthesis approaches have also been used for the production of glyco-diversified NPs. Unfortunately, these methods are not eco-friendly and require multiple time-consuming steps with low final-product yields. Enzymatic glyco-diversification of NPs requires pure enzymes and expensive NDP-sugar donors that limits the scale-up process and makes the purification process tedious and time-consuming. Thus, generating a robust microbial host platform for efficient biosynthesis of diverse sugars by simple fermentation is a highly attractive approach for industrial purposes. Several reports have shown production of diverse NP-glycosides in practical quantities (Pandey et al., 2016). Moreover, such microbial systems could also be engineered to utilize different carbon sources derived from natural biomass (Zhang et al., 2015; Zhou et al., 2015). Development of such NP-engineering microbial systems that can utilize renewable resources offers a sustainable way for the production of a diverse array of functional compounds at a low cost. The *de novo* biosynthesis of such complex molecules can also be achieved by stable co-culture and polyculture systems (Zhou et al., 2015; Jones et al., 2017).

Naturally occurring therapeutics, cosmetics, and nutraceuticals in current use have a diverse set of sugars in their structures. For example, vancomycin, erythromycin, doxorubicin, and amphotericin B are selected microbial secondary metabolites that are decorated with a diverse set of highly modified sugar units. The removal or alteration of the sugar units results in a change in the physicochemical and biological properties of these compounds (Weymouth-Wilson, 1997). In most cases, glycoside molecules lose the biological potential upon release of the sugar unit from the parent molecule. Thus, sugar units are the essential active parts of therapeutic molecules that usually play a pivotal role in recognition of the target site and/or even the molecular mechanism of action. Similarly, in cosmetics and dietary supplements, conjugation of sugar appendages enhances the water solubility, bioavailability, and stability of the molecules. Thus, sugar residues serve as a key platform for the development of novel molecules.

In another aspect, glycosylating microbial platforms could also be utilized to detoxify pollutants, pesticides, and xenobiotics from the environment. Evidence shows that toxic molecules usually turn to non-toxic molecules after conjugation of the sugar units (Stupp et al., 2013; Parajuli et al., 2016; Thierbach et al., 2017). Thus, engineered sustainable glycosylating microbial platforms could be a next generation system for remediation by removing pesticides and toxic molecules from the environment and industrial waste.

In conclusion, engineered microbe-mediated exchange of microbial glycone parts with exogenously supplemented NPs is a fascinating approach to accelerate the production of novel molecules for use in human beings. Such systems can also be extended to waste treatment and detoxification of the environment. To achieve these goals, the system should be highly sustainable and robust. Nevertheless, for a high-titer production of NP-glycosides by simple fermentation using engineered microbes as cell factories, engineering microorganisms with recently developed systems/synthetic biology tools is essential while generating promiscuous GTs by mutagenesis.

## Author contributions

The author confirms being the sole contributor of this work and approved it for publication.

### Conflict of interest statement

The author declares that the research was conducted in the absence of any commercial or financial relationships that could be construed as a potential conflict of interest.
